# Knowledge, Attitude, and Practice Related to Hemovigilance: A Cross-Sectional Study Among Entry-Level Medical Professionals in a Tertiary Care Teaching Hospital in Northern India

**DOI:** 10.7759/cureus.104006

**Published:** 2026-02-20

**Authors:** Moumita Bala, Ekta Arora, Shouvik Choudhury, Shoma Mukherjee

**Affiliations:** 1 Department of Pharmacology, School of Medical Sciences and Research, Sharda University, Greater Noida, IND; 2 Department of Pharmacology, Government Institute of Medical Sciences, Greater Noida, IND; 3 Department of Pharmacology, Burdwan Medical College, Burdwan, IND

**Keywords:** adverse transfusion reactions, attitude, blood transfusion, hemovigilance, knowledge, practice

## Abstract

Background: Hemovigilance is a critical surveillance system designed to enhance blood transfusion safety by monitoring, detecting, and preventing adverse transfusion reactions. Despite the launch of the Hemovigilance Program of India, awareness and engagement remain notably low among entry-level medical professionals, potentially compromising patient safety and quality of care.

Objective: This study aimed to assess the knowledge, attitude, and practice (KAP) related to hemovigilance, identify barriers to adverse transfusion reaction reporting, and recommend strategies to enhance participation and improve reporting culture among entry-level medical professionals at a tertiary care teaching hospital in northern India.

Methods: A descriptive, cross-sectional questionnaire-based survey was conducted among 414 entry-level medical professionals, including MBBS students (first & second professional years), postgraduate medical trainees, and resident doctors. The 20-item questionnaire explored various domains such as demographic characteristics, knowledge related to hemovigilance, and attitudes toward transfusion reaction reporting, actual reporting practices, perceived barriers, and potential strategies to enhance reporting. Data were collected in offline mode over a period of three months using a pen-and-paper approach.

Results: The majority of participants, 376 (90.8%), were between 20 and 30 years of age, with a predominance of female participants, 234 (56.5%). Awareness of transfusion reactions was high (386; 93.2%), and most participants recognized their preventability (328; 79.2%), yet only 152 (36.7%) were aware of the National Hemovigilance Program. Positive attitudes towards hemovigilance were evident, with nearly all participants endorsing its benefits (414; 100%) and acknowledging professional responsibility to report (396; 95.6%). Support for curricular integration (402; 97.1%) and enrolment of every institution under PvPI (384; 92.7%) was strong. However, only 56 (13.5%) had witnessed a transfusion reaction, and a meagre 22 (5.3%) had reported one, signifying a substantial practice gap. Suggested improvements included user-friendly mobile app development, compulsory reporting, and targeted educational interventions.

Conclusion: Although medical professionals demonstrated strong knowledge and positive attitudes toward hemovigilance, practical implementation is lacking. Enhanced education, streamlined reporting tools, and institutional support are imperative to bridge the gap between awareness and practice, thereby improving blood transfusion safety and patient outcomes. Early introduction of hemovigilance and incentivization like certificates of appreciation, acknowledgement of names on hospital display boards or newsletters/magazines, grace marks, attendance credit for students, and a token of appreciation for faculty members can play a big role in improving reporting.

## Introduction

Blood and blood product transfusion plays a vital role in improving patient outcomes in various acute and chronic medical conditions. While blood (& products) transfusion is lifesaving [1], transfusion reaction can limit its utility by causing immediate harm to the patient, necessitating prolongation of hospitalization and further adding to healthcare costs. Transfusion reaction can be defined as any transfusion having a certain risk and any unfavorable event occurring in the patient during or after transfusion, for which no other reason can be found [2]. Blood transfusion often carries a significant risk of adverse transfusion reactions, which can range in severity from mild to life- threatening with an incidence (in published reports) between 0.2 and 10% globally and around 0.084% in India [3,4]. This estimation rate in India can be an underestimation of the true incidence due to limited data and a lack of awareness and reporting of transfusion-related adverse events. Hemovigilance thus acts as a vital sentinel system in blood transfusion, continuously monitoring and assessing the safety of blood and blood products throughout the transfusion chain. It includes methods for identifying errors, adverse events, and reactions, including alert systems, investigation of complaints, traceability systems, notification systems, and audits of practice [1].

The word ‘hemovigilance’ was first coined in France in 1991 in analogy to the already existing term ‘pharmacovigilance’, derived from the Greek word ‘haema’ means blood, and the Latin word ‘vigilance’ means watchful. Further, the International Hemovigilance Network has defined Hemovigilance as “a set of surveillance procedure covering the whole transfusion chain from the collection of blood and its components to the follow-up of its recipients, intended to collect and assess information on unexpected or undesirable effects resulting from the therapeutic use of labile blood products and to prevent their occurrence or recurrence” [5-9].

The Indian Pharmacopeia Commission (IPC), under the aegis of the Ministry of Health and Family Welfare, Government of India, in collaboration with the National Institute of Biologicals, Noida, thus launched the Hemovigilance Program in India (HvPI) on 10th December 2012 as an integral part of the Pharmacovigilance Program of India (PvPI). It is a centralized structured program that coordinates various activities in the country between blood banks, transfusion services, hospitals, healthcare, regulatory agencies, and national health authorities [10].

Hemovigilance, though proven to be an effective tool for improving transfusion practice and patient safety, is not yet practiced widely in Indian hospitals [11]. Considering that blood transfusion can be both a boon and a bane, knowledge of preventing harm during patient treatment and care is of utmost importance. Hemovigilance remains an unmet need in India due to underreporting of adverse transfusion events, lack of training among healthcare professionals (HCPs), and infrastructural limitations (particularly in rural India).

Reporting transfusion reactions is of primary importance for the success of the Hemovigilance Program of a country for generating data on adverse transfusion reactions. The information thus collected would facilitate correction and preventive actions to be taken to minimize the potential risks associated with blood collection processing and transfusion to patients.

While participation in the Hemovigilance Program has expanded over the years, inadequate awareness and underreporting of transfusion-related adverse events continue to pose challenges. Addressing these gaps is crucial for effective identification of reactions and for ensuring safe and rational use of blood and blood products.

In the absence of a robust hemovigilance system, accurate assessment of transfusion-associated risks becomes difficult, thereby limiting the ability of HCPs, particularly those at the entry level of their medical career to appropriately evaluate the benefit-risk ratio and counsel patients prior to transfusion. Strengthening the knowledge, attitudes, and practices (KAP) of medical professionals regarding the identification and reporting of adverse transfusion reactions from the earliest stages of their careers is therefore essential. Accordingly, the present study was undertaken to assess the KAP related to hemovigilance, to identify self-perceived barriers to report transfusion-related events among entry-level medical professionals, who are likely to play a pivotal role in the future of transfusion safety monitoring. The findings of this study are expected to support evidence-based recommendations aimed at strengthening institutional hemovigilance programs, improving reporting compliance, and ultimately enhancing patient safety in transfusion practices.

## Materials and methods

Study design

This study employed a descriptive, cross-sectional, questionnaire-based design and was conducted at a tertiary care teaching hospital in Northern India. The target population comprised entry-level medical professionals. “Entry-level” medical professionals may be defined as medical students/professionals at the initial stage of their medical training or career who have limited but developing exposure to patient care, clinical procedures, and healthcare systems. They are typically in the foundational phase of acquiring essential knowledge, skills, and attitudes required for independent medical practice. Thus, in this study, entry-level medical professionals comprised undergraduate medical students, postgraduate trainees, and resident doctors. The undergraduate group included first-year MBBS students, those who had exposure to foundation courses or early clinical exposure (ECE) programs, as well as second-year MBBS students engaged in pre-clinical and para-clinical postings. The postgraduate cohort consisted of first-year MD/MS trainees who had recently initiated specialty training and possessed limited clinical experience. In addition, resident doctors included tutors with less than one year of experience in academic and clinical responsibilities.

Sample size calculation

The sample size was calculated using the Raosoft software (http://www. Raosoft.com/sample size). The required sample size was estimated at the 95% confidence level, with an estimated 50% prevalence rate and a margin of error of 5%. The required minimum sample size was determined to be 249. A random sampling technique was employed to recruit participants, ensuring the inclusion of available and willing participants at the time of data collection.

Questionnaire development

A structured and pre-validated questionnaire was used as the primary tool for data collection (see the Appendices). The questionnaire was formulated with reference to similar studies conducted earlier [11-13]. The validation process was carried out through a pilot study involving similar respondents to those in the main study. The questionnaire consisted of 20 items designed to gather information across several domains, including demographic details, knowledge, attitudes, practices, perceived barriers, and strategies to enhance hemovigilance reporting. It incorporated both open-ended and closed-ended questions to elicit comprehensive quantitative and qualitative responses from participants.

Data collection

Data collection was carried out using a structured, predesigned questionnaire administered in offline (pen-and-paper) mode. Prior to initiation of the study, four tutors/resident doctors were trained regarding the objectives of the study, eligibility criteria, standardized questionnaire administration, and procedures for data handling to ensure uniformity and minimize inter-observer variability.

Data collection was conducted over a period of 3 months from November 2024 to January 2025, where the trained team of tutors visited various In-Patient and Out-Patient Departments, and eligible participants were approached in person, as per the Duty Roster, excluding all holidays. The objectives and nature of the study were explained to them in detail before questionnaire distribution. After the participant voluntarily consented to participate in this study, adequate time was provided to each participant to complete the questionnaire independently to reduce response bias. The data collectors remained available to clarify procedural doubts, if any, without influencing participants’ responses.

Completed questionnaires were checked on-site for completeness and legibility. Incomplete or partially filled questionnaires were excluded from the final analysis. Collected data were subsequently categorized, coded, and tabulated in a standardized format prior to statistical analysis.

Data analysis

Data obtained were tabulated in an Excel spreadsheet (Microsoft Corporation, Redmond, Washington, United States), and a thorough quality check was performed on the compiled data. A manual cross-check was also conducted to ensure data reliability. Descriptive statistics, including frequencies and percentages, were used to summarize the findings.

Ethical considerations

The study was conducted in accordance with the ethical principles outlined in the Declaration of Helsinki and received approval from the Institutional Ethics Committee of the School of Medical Sciences and Research & Sharda Hospital (Ref No: SU/SMS&R/76-A/2023/163). All procedures complied with established ethical standards as per GCP guidelines, to ensure the protection of the participants' rights and confidentiality. Informed consent was obtained from all participants prior to filling out the questionnaire.

## Results

Out of the total 508 questionnaires administered to participants, 414 were duly filled and returned, thus giving a response rate of 81.5%.

Socio-demographic variables

The age distribution indicated that most participants, 376 (90.8%), were between 20 and 30 years of age, with a mean age of 23 ± 4 years. The gender distribution showed a predominance of females, with 234 participants (56.5%), indicating a slightly higher representation of women in the study population. Based on eligibility criteria, the analysis showed that most participants were from the 1st and 2nd professional years, comprising 128 and 164 students (30.9% and 39.6%, respectively). Additionally, 98 participants (23.7%) were postgraduate medical trainees, and 24 (5.8%) were resident doctors (Table 1).

**Table 1:** Socio-demographic variables

Variables		n (%)
Age in years	<20	26 (6.3)
	20-30	376 (90.8)
	>30	12 (2.9)
Gender	Male	180 (43.5)
	Female	234 (56.5)
	Other	00
Educational qualification	1^st^ Prof students	128 (30.9)
	2^nd^ Prof students	164 (39.6)
	Postgraduate Medical Trainees	98 (23.7)
	Resident doctors	24 (5.8)

Knowledge of hemovigilance among participants

The analysis of knowledge related to hemovigilance among the study participants revealed that 386 (93.2%) were aware of the occurrence of transfusion reactions, indicating a high level of basic understanding of the subject, followed by 328 (79.2%) who were aware that blood transfusion reactions can be prevented. However, only 208 participants (50.2%) understood that not just severe reactions, but all transfusion reactions, need to be reported. Furthermore, 190 (45.9%) knew who all can report transfusion reactions, and only 152 (36.7%) were aware of the existence of the Hemovigilance Program of India (Table 2).

**Table 2:** Knowledge of hemovigilance among participants

No.	Variables	n (%)
1	Knowledge about transfusion reactions?	386 (93.2)
2	Awareness about prevention of blood transfusion reactions?	328 (79.2)
3	Know that only severe transfusion reaction needs to be reported?	208 (50.2)
4	Know who can report transfusion reaction?	190 (45.9)
5	Awareness about Hemovigilance Program of India?	152 (36.7)

Attitude towards hemovigilance

The majority of participants demonstrated a positive attitude toward hemovigilance and transfusion reaction reporting. Reporting was perceived as beneficial by 414 (100%) participants, and 392 (95.6%) recognized it as a professional responsibility. Inclusion of hemovigilance in the undergraduate curriculum was supported by 402 (97.1%) participants, while 384 (92.7%) endorsed institutional enrolment in hemovigilance programs. Additionally, 402 (97.1%) emphasized the importance of educating blood bank staff (Table 3).

**Table 3:** Attitude towards hemovigilance

No.		Agree n (%)	Strongly agree n (%)	Disagree n (%)	Strongly disagree n (%)	Undecided n (%)
1	Reporting of transfusion reaction is beneficial	250 (60.4)	164 (39.6)	00	00	00
2	Reporting of transfusion reaction is a professional duty	218 (52.6)	178 (43)	2 (0.5)	00	16 (3.9)
3	Hemovigilance should be taught in undergraduate curriculum to inculcate the habit of reporting	254 (61.4)	148 (35.7)	00	00	12 (2.9)
4	Every medical institute should get enrolled under Hemovigilance Program	212 (51.2)	172 (41.5)	12 (2.9)	00	18 (4.4)
5	Blood Bank staff should be educated and can play a pivotal role in reporting	178 (43)	224 (54.1)	6 (1.4)	00	6 (1.4)

Practice among individuals regarding hemovigilance

**Figure 1:**
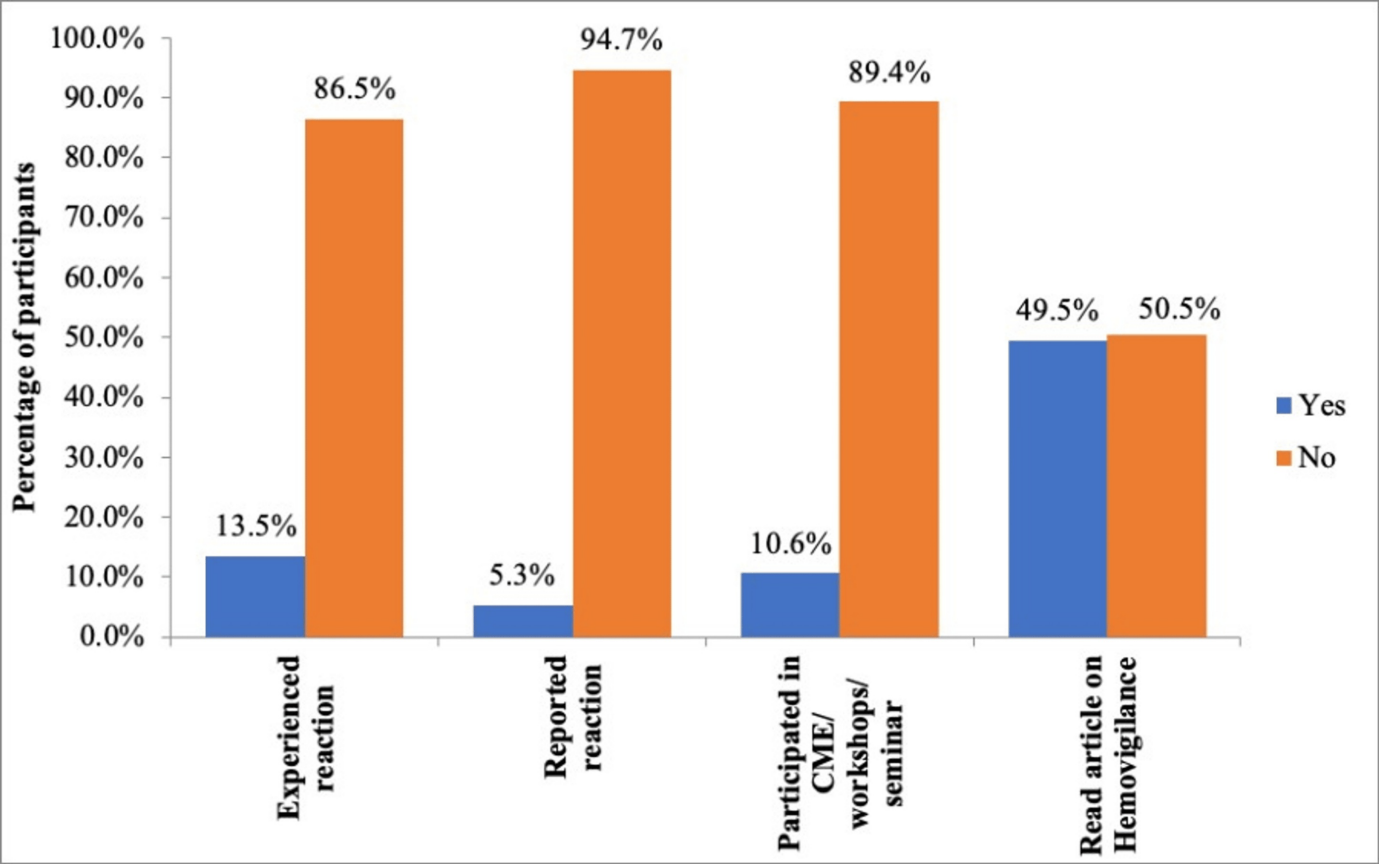
Practice among individuals regarding hemovigilance

The analysis of practices related to hemovigilance among the participants revealed that only 56 (13.5%) had witnessed a transfusion reaction. Among those who witnessed a transfusion reaction, only 22 (5.3%) reported it, while 392 (94.7%) did not report any reaction. Regarding educational exposure to hemovigilance, the majority of participants, 370 (89.4%), had not attended any continuing medical education (CME) sessions or training, and 209 (50.5%) had not read any study material related to hemovigilance (Figure 1).

Self-perceived limitations and strategies for improvement

The responses regarding strategies to improve transfusion reaction reporting highlighted several key recommendations from the study participants. The majority of participants, 180 (43.5%), advocated for the development of a mobile application compatible with other phones apart from Android, to streamline the reporting process. Additionally, 68 (16.4%) participants suggested launching a user-friendly helpline to further facilitate unhindered reporting. Training HCPs in reporting practices was emphasized by 72 (17.4%) participants, while 42 (10.1%) recommended making transfusion reaction reporting mandatory to ensure compliance. 38 (9.2%) participants proposed organizing CME sessions, workshops, or seminars to enhance knowledge and raise awareness. Simplifying the reporting process was suggested by six (1.5%) participants, and eight (1.9%) emphasized the importance of ensuring the availability of transfusion reaction reporting forms in the wards to encourage timely and accurate reporting (Table 4).

**Table 4:** Questions based on self-perceived limitations and strategies for improvement CME: Continuing medical education

Self-perceived limitations and strategies for improvement		n (%)
1	Ensure the availability of transfusion reaction reporting forms in all wards	8 (1.9)
2	Simplify the process of transfusion reaction reporting	6 (1.5)
3	Participating in CME/workshops/seminar	38 (9.2)
4	Making reporting mandatory	42 (10.1)
5	Healthcare professionals should receive training on reporting	72 (17.4)
6	Launching of a user-friendly helpline number	68 (16.4)
7	Development of mobile application (other than android)	180 (43.5)

Comparison of knowledge vs practice scores among different participants

The mean knowledge score among 1st Professional students (n = 128) was 2.84 ± 0.88, compared to 3.08 ± 0.99 among 2nd Professional students (n = 164), with no statistically significant difference observed (p = 0.09). Postgraduate medical trainees had a higher mean knowledge score (3.93 ± 0.95) than resident doctors (3.33 ± 0.96), and this difference was statistically significant (p = 0.01).

In terms of practice scores, 1st Professional students had a mean score of 0.58 ± 0.54, while 2nd Professional students had a mean score of 0.83 ± 0.80; this difference was statistically significant (p = 0.02). The mean practice score among postgraduate medical trainees was 1.14 ± 0.94, compared with 0.79 ± 0.59 among resident doctors; however, this difference was not statistically significant (p = 0.11) (Table 5).

**Table 5:** Comparison of knowledge vs practice scores among different participants

Participants	Number (n)	Knowledge score (Mean ± SD)	p value	Practice score (Mean ± SD)	p-value
1^st^ Prof students	128	2.84 ±0.88	0.09	0.58 ± 0.54	0.02
2^nd^ Prof students	164	3.08 ± 0.99		0.83 ±0.80	
Postgraduate medical trainees	98	3.93 ± 0.95	0.01	1.14 ± 0.94	0.11
Resident doctors	24	3.33 ± 0.96		0.79 ± 0.59	

## Discussion

Hemovigilance plays an important role in tracking and reducing the adverse events related to blood transfusions and blood products by seeking the active involvement of HCPs [14]. Despite the emphasis on reporting adverse reactions related to blood and blood products, the reporting rates in India remain significantly below expectations. To address this gap, the present study was undertaken with the aim of assessing the KAP related to transfusion reactions. Additionally, the study aimed to identify the reasons for underreporting and explore potential strategies to enhance the reporting of transfusion reactions within our tertiary care teaching institution.

Socio-demographic findings from the current study suggest that the majority of participants were undergraduate students, particularly in their early years of professional education, with a relatively smaller number of participants comprising postgraduate medical trainees.

The current study reveals that the majority of the participants demonstrated knowledge of the occurrence of transfusion reactions and how they could be prevented. Another study has also reported similar findings, where around 85% of the participants had the correct understanding of hemovigilance [15]. However, a notable deficiency was noted in the comprehensive knowledge regarding hemovigilance, especially, many are unaware of the imperative to report all transfusion-related adverse events, the roles and responsibilities in the reporting process, and the existence and objectives of the Hemovigilance Program of India. The findings from a study conducted in Nashik, Maharashtra aligns with this observation, where 58% of HCPs exhibited poor knowledge of hemovigilance [11]. These findings highlight the need for targeted educational initiatives to improve awareness of hemovigilance systems and the reporting mechanisms, which are essential for enhancing patient safety and monitoring transfusion-related events in our institution.

The attitude assessment of the participants demonstrated a strong consensus on the importance of reporting transfusion reactions and the role of hemovigilance in improving patient safety. Most participants recognized reporting as both beneficial and a professional duty, indicating a positive attitude towards improving transfusion safety practices. Most of the participants believed that hemovigilance should be integrated into the undergraduate curriculum from the 1st year onward, as ECE may help inculcate the habit of reporting and promote a culture of vigilance and patient safety among our future professionals. The main objective of ECE is to create an opportunity for students to correlate learning in Phase I subjects with their clinical application. The aim of ECE is not only to strengthen the foundational knowledge of students but also to ensure that this learning is carried forward into later phases of their training, ultimately helping them develop into stronger and more competent HCPs. As a part of the ECE, they should receive basic teaching on drug safety. While they learn extensively about the therapeutic benefits of drugs, it is equally important to sensitize them to the adverse drug reactions to drugs & blood products. This early awareness will help them develop a balanced understanding of both the efficacy and risks of pharmacological interventions.

In the second year, the PvPI module is already incorporated into the CBME curriculum, and since hemovigilance is an integral component of PvPI, students can be taught about these concepts in a more comprehensive and detailed manner. At this stage, they begin regular clinical postings, interact more closely with patients, and actively participate in patient care. Such enhanced clinical exposure enables them to better appreciate the importance of recognizing transfusion reactions, preventing them, and understanding how the Hemovigilance Program of India contributes to patient safety.

The majority agreed that medical institutions should enroll in the Hemovigilance Program and acknowledged that blood bank staff can play a pivotal role in effective reporting practices. Similar results have been reported by earlier studies as well [11,16]. These findings highlight the need for major changes, including educational reforms, institutional participation, and focused training for blood bank staff, to enhance hemovigilance practices and foster a culture of safety and accountability.

The participants' practices reveal notable deficiencies in the implementation of hemovigilance. Although a small proportion of individuals did encounter transfusion reactions, the reporting rates were notably low, indicating a lack of adherence to reporting protocols or insufficient awareness of their importance. The majority of participants had not attended any CME sessions or training related to hemovigilance. The limited participation in CME sessions and training programs further underscores the need for targeted educational initiatives to enhance knowledge and skills in hemovigilance. While approximately half of the participants had read articles on hemovigilance, the other half lacked exposure to relevant literature, suggesting a need to promote access to educational resources. But a striking behavior of the participants showed that even though nearly 50 percent claimed to have read articles on hemovigilance, they still didn’t report. A recent study also found that only 18% participants had attended CME/workshops on hemovigilance [11,16-17]. These results point to a pressing need for structured training, increased dissemination of hemovigilance materials, and reinforcement of reporting protocols to improve compliance and strengthen hemovigilance practices in the country. While exploring strategies to improve the reporting of transfusion reactions, participants demonstrated a strong preference for innovative and practical approaches to enhance the reporting process. The development of a mobile application compatible with all phone models and not just Android phones was the most favored suggestion, reflecting the need for user-friendly and accessible digital tools to simplify reporting. The importance of training HCPs and organizing educational initiatives, such as CMEs and workshops, was also emphasized. Other recommendations, such as making reporting compulsory, highlight the need for systemic and structural changes to improve compliance. These results suggest that a multifaceted approach, combining digital solutions, education, and institutional reforms, is essential to strengthen hemovigilance practices and ensure effective reporting of transfusion reactions. Moreover, leveraging digital tools, such as mobile applications and automated reporting systems, as recommended in recent studies [12], can play a transformative role in improving the efficiency and accuracy of transfusion reaction reporting. To improve the quality and frequency of hemovigilance reporting, we recommend simplifying the Transfusion Reaction Reporting process to make it easier and quicker for HCPs to complete. Streamlined, user-friendly forms will reduce the documentation burden and encourage timely submissions. Additionally, implementing a QR-code-based reporting system across hospital wards, ICUs, emergency units, and blood banks will enable HCPs to instantly report adverse reactions to blood and blood products. This will eliminate the need to download forms manually, simplify the reporting process, and significantly improve real-time vigilance. Many studies also recommended that incentives can improve reporting [14]. Incentives can include certificates of appreciation, acknowledgement of names on hospital display boards or newsletters/magazines, grace marks, attendance credit for students, and a token of appreciation for faculty members.

The findings from the current study indicate that while there were significant differences in knowledge scores among the groups, practice scores did not show a similar trend. The postgraduate medical trainees demonstrated better knowledge compared to their counterparts, suggesting that the foundational stage of education may contribute to heightened awareness. However, the lack of significant differences in practice scores highlights a disconnect between knowledge and its application, emphasizing the need to bridge this gap. Similar results have been reported by other studies involving HCPs and interns [12,18].

Educational initiatives should focus not only on improving knowledge but also on translating it into better hemovigilance practices through hands-on training, mentorship, and reinforcement of reporting protocols.

Limitations

 Being a single-center study, the findings may not be generalizable to other institutions with different patient loads, training structures, or transfusion practices. The cross-sectional design captures knowledge and practices at a single point in time and does not allow assessment of changes over time. In addition, data were collected using a self-administered questionnaire, which may be subject to recall bias and social desirability bias. Although key categories of healthcare personnel involved in transfusion practices were included, nursing staff and paramedical personnel were not part of the study, which may limit the comprehensive assessment of hemovigilance practices within the institution.

Recommendations

We propose several recommendations to strengthen reporting practices. Incentive-based strategies may motivate HCPs to actively report adverse reactions related to blood and blood products, with high-frequency reporters being acknowledged through certificates of appreciation on a monthly basis. Formal recognition of contributors through hospital display boards, newsletters, magazines, or institutional journals may further reinforce the value of reporting. In addition, tailored incentives for students and faculty could be considered; students may be encouraged through academic benefits such as bonus attendance or grace marks for consistent reporting, while faculty members may be supported through compensatory benefits or other appropriate forms of acknowledgment. Alongside educational interventions, such incentive-driven approaches have the potential to substantially improve engagement and foster a robust culture of reporting across our center.

## Conclusions

While HCPs exhibited strong knowledge and a positive attitude toward hemovigilance, their practice scores were notably lower. The findings indicate that, despite adequate awareness and favorable attitudes, there is a significant gap in implementing hemovigilance practices. To bridge this gap and enhance reporting, emphasis should be placed on additional training programs, simplifying reporting process, developing a user-friendly mobile application, and organizing more CME sessions and workshops. Furthermore, incentivization can also go a long way. Incentives can include certificates of appreciation, acknowledgement of names on hospital display boards or newsletters/magazines, grace marks, attendance credit for students, and a token of appreciation for faculty members. Hence, in addition to educational interventions, incentivization can significantly enhance participation and improve the overall reporting culture at our center.

## Appendices

Questionnaire on hemovigilance

Name of the participant (initials only):

Age:

Gender:

Qualification: 

Tick the relevant details: (1st Prof. MBBS, 2nd prof. MBBS, Postgraduate Medical Trainees, Resident doctors)

**Table 6:** Knowledge regarding hemovigilance

No.	Variables	YES	NO
1	Knowledge about transfusion reactions?		
2	Awareness about prevention of blood transfusion reactions?		
3	Know that only severe transfusion reaction needs to be reported?		
4	Know who can report transfusion reaction?		
5	Awareness about Hemovigilance Program of India?		

**Table 7:** Attitude towards hemovigilance

No.		Agree	Strongly agree	Disagree	Strongly disagree	Undecided
1	Reporting of transfusion reaction is beneficial					
2	Reporting of transfusion reaction is a professional duty					
3	Hemovigilance should be taught in undergraduate curriculum to inculcate the habit of reporting					
4	Every Medical Institute should get enrolled under Hemovigilance Program					
5	Blood Bank staff should be educated and can play a pivotal role in reporting					

**Table 8:** Practice regarding hemovigilance

No.		YES	NO
1	Have you come across any transfusion reactions during your professional practice?		
2	Have you ever reported any transfusion reaction to the hemovigilance center?		
3	Have you participated any CME Programs/workshops/seminar’s on hemovigilance?		
4	Have you read any article for prevention of transfusion reaction?		

**Table 9:** Factors that discourage reporting transfusion reactions

No.		YES	NO
1	Do you think lack of knowledge about how to report and where to report discourages you to report transfusion reaction? If yes what are the other factors:		
I	Concern regarding Legal liability		
II	Shortage of time for reporting		
III	Only blood banks are authorized to report		
IV	Difficulty in determining whether a transfusion reaction has occurred or not?		
V	Lack of recognition of hard work		
VI	Individual reporting will not make any significant difference to the database		
VII	Don’t find it necessary to report		
VIII	Any other:		

**Table 10:** Potential strategies to enhance transfusion reaction reporting

1	Ensure the availability of transfusion reaction reporting forms in all wards	
2	Simplify the process of transfusion reaction reporting	
3	Participating in CME/workshops/seminar	
4	Making reporting mandatory	
5	Health care professional should receive training on reporting	
6	Launching of a user friendly toll-free helpline number	
7	Development of mobile application (other than android)	
